# Supplementary material for: Exploiting lung adaptation and phage steering to clear pan-resistant Pseudomonas aeruginosa infections in vivo
Source: Nat Commun. 2024 Feb 20;15:1547. doi: 10.1038/s41467-024-45785-z (PMC10879199; doi:10.1038/s41467-024-45785-z)
Supplement: Supplementary file 1 — Supplementary Information [file 41467_2024_45785_MOESM1_ESM.pdf]

Supplementary Table 1. Description of bacteriophages included in the cocktail.

Bacteriophage	Origin	Morphology	Target	Sequence Accession Number	Reference
PELP20	Tbilisi, Georgia	Myoviridae	LPS	PRJEB67471	Essoh et al, 2013
14/1	Regensburg, Germany	Myoviridae	LPS	NC_011703	Merabishvili et al, 2009
PT6	Tbilisi, Georgia	Podoviridae	Type IV Pilus	n/a	Ceyssens et al, 2011
PNM	Tbilisi, Georgia	Podoviridae	Type IV Pilus	PRJEB67471	Merabishvili et al, 2009

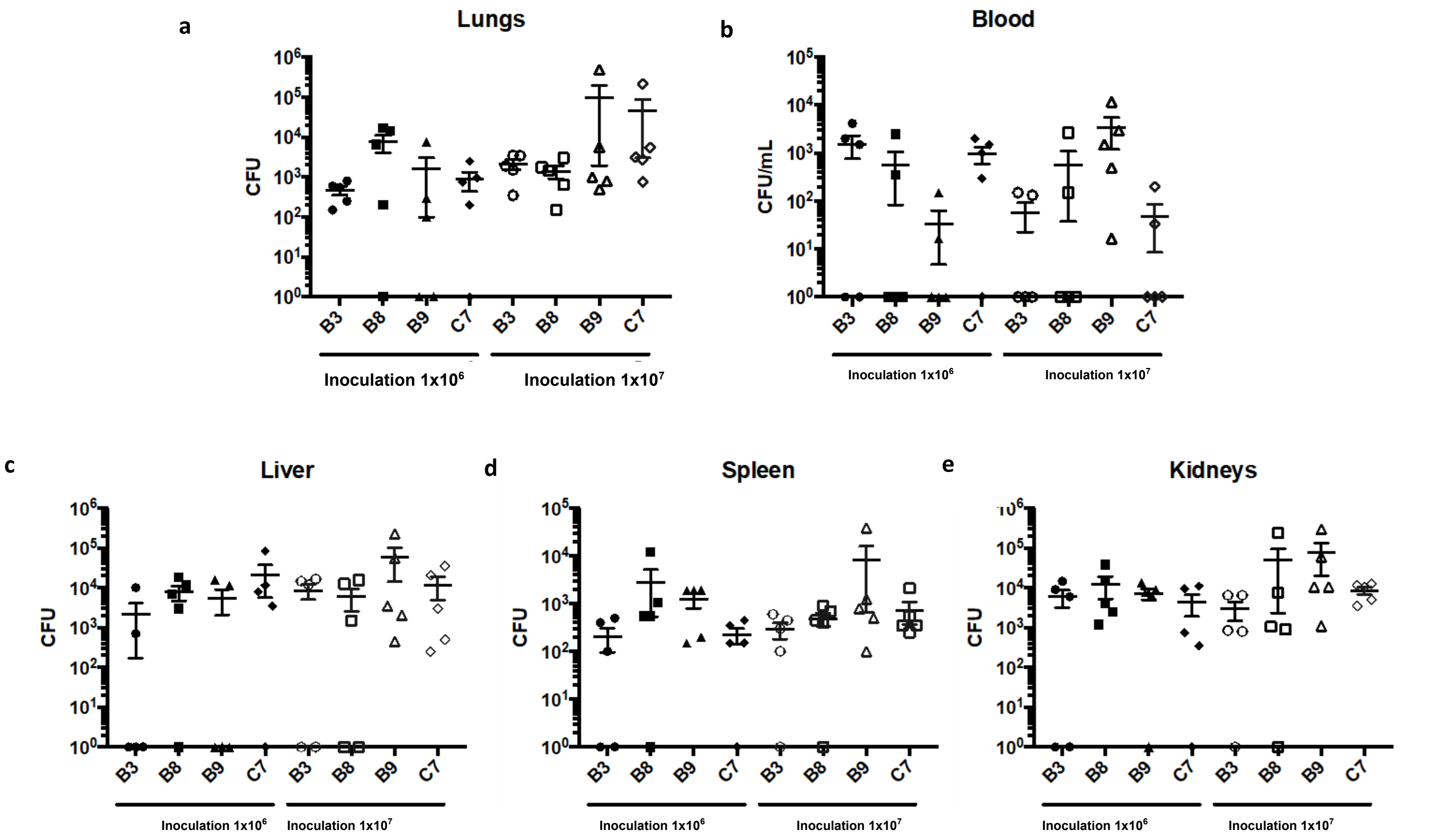

Supplementary Figure 1. BALB/c mice infected with four different strains of multidrug resistant *P aeruginosa*. Bacterial load was determined in the a) lung b) blood c) liver d) spleen e) kidneys at 48hrs post-infection. The mean and SEM is indicated, N=5 mice per group, 1 independent experiment. The y-axis has been corrected by adding 1 (to zero all samples). Each symbol represents the bacterial numbers in individual mice. Circles represent strain B3, squares represent strain B8, triangles represent strain B9 and diamonds represent strain C7. The inoculation dose of $1x10^6$ is indicated by black symbols and the inoculation dose of $1x10^7$ is indicated by white symbols. Source data are provided as a Source Data file.

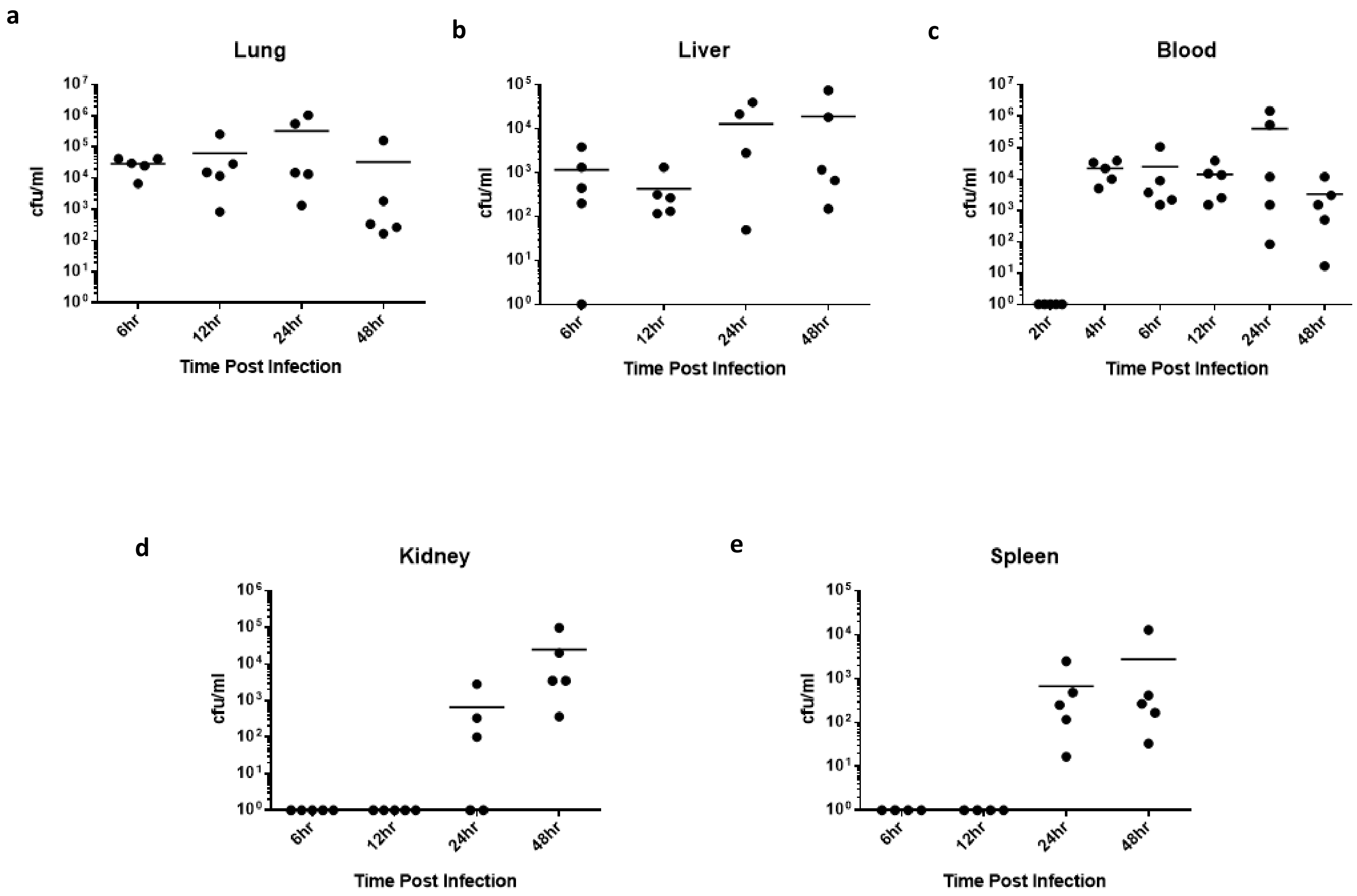

Supplementary Figure 2. *P. aeruginosa* systemic infection model.

Shows the bacterial numbers present in BALB/c mice after intranasal *P. aeruginosa* at 1 x 10^6 cfu/ml in the a) lungs, b) liver, c) blood, d) kidney and e) spleen. The mean is indicated, with n=5 mice per group per timepoint, 1 independent experiment. The y-axis has been corrected by adding 1 (to zero all samples). Each black circle represents the bacterial numbers in individual mice. Source data are provided as a Source Data file.

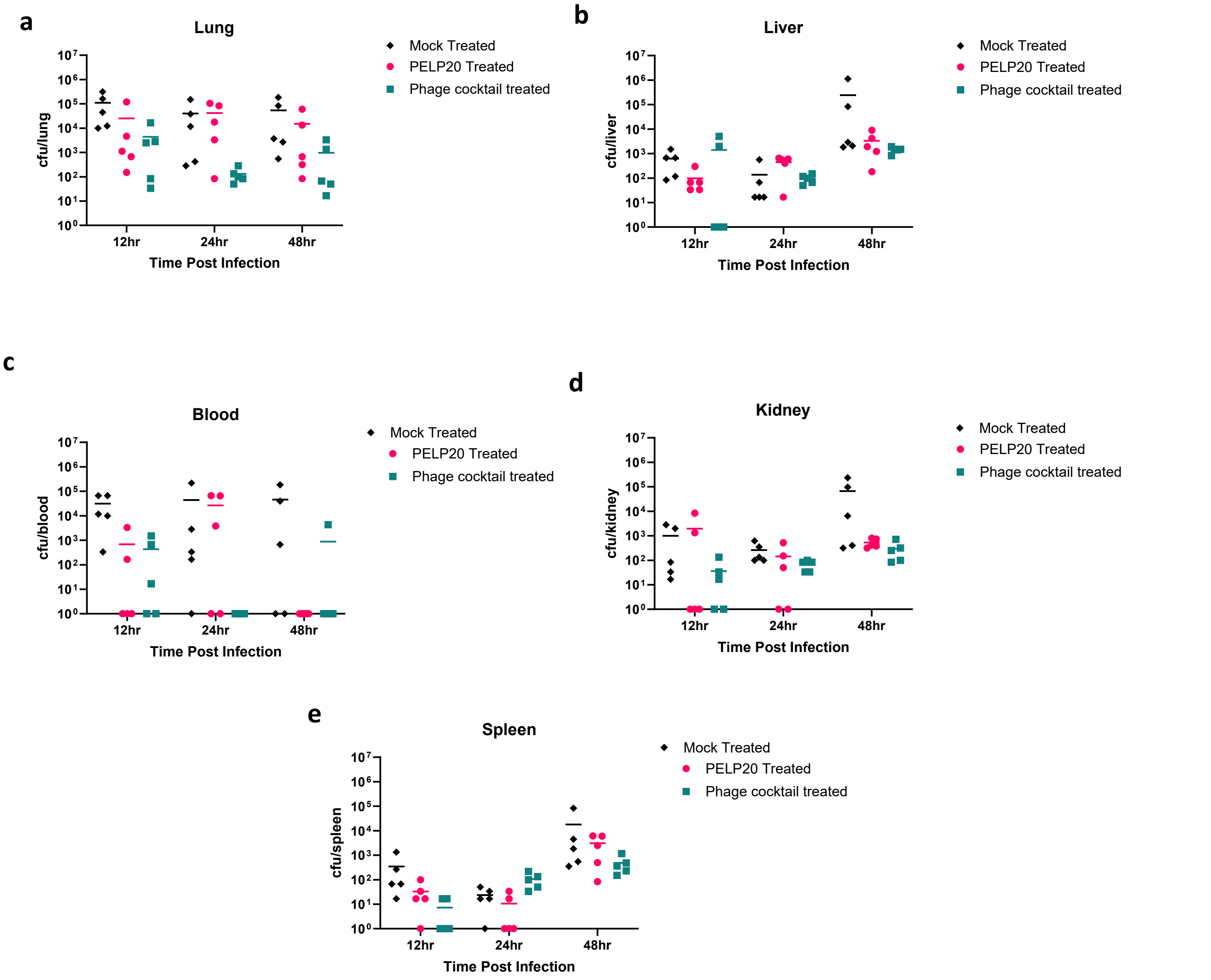

Supplementary Figure 3. Delayed Intranasal Treatment *in vivo* using phage. Shows the bacterial numbers after treatment in the a) lungs b) liver c) blood d) kidney e) spleen. Mice infected with 1x10^{6} cfu/ml *P. aeruginosa* were treated intranasally with PBS (black diamonds), treated intranasally with phage PELP20 5hr after infection (pink circles) or treated intranasally with a phage cocktail 5hr after infection (green squares). The mean is indicated, with n=5 mice per group per timepoint, 1 independent experiment. The y-axis has been corrected by adding 1 (to zero all samples). Statistics were performed to compare the mock treated and phage treated groups via two-way ANOVA with Bonferroni correction post hoc test was performed when comparing more than 3 experimental groups. Source data are provided as a Source Data file.

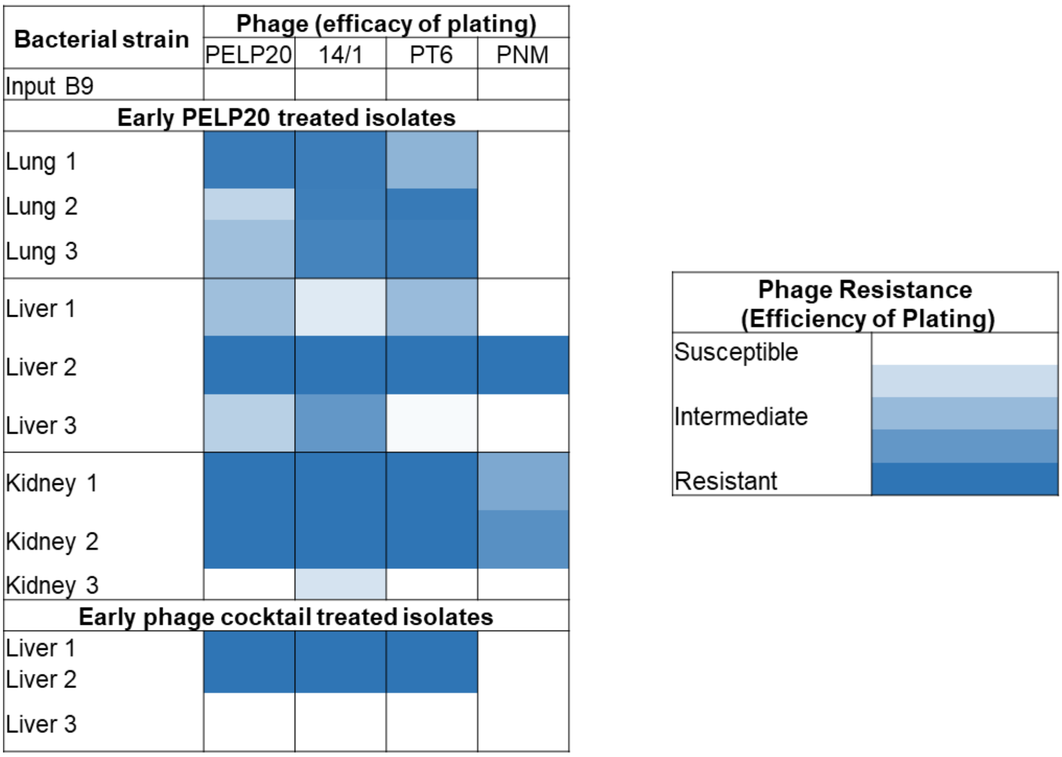

Supplementary Figure 4. Phage Resistance of Early Phage Treated Isolates Recovered From Systemic infection *In Vivo* Model.

Shows heat map showing the phage resistance (via efficacy of plating) of isolates recovered from the early phage treated mice to the phages present in the cocktail, n= 3 isolates tested for each group. Phage resistance is represented by dark blue squares, and phage susceptibility is represented by white squares. Source data are provided as a Source Data file.

a

b

c

d

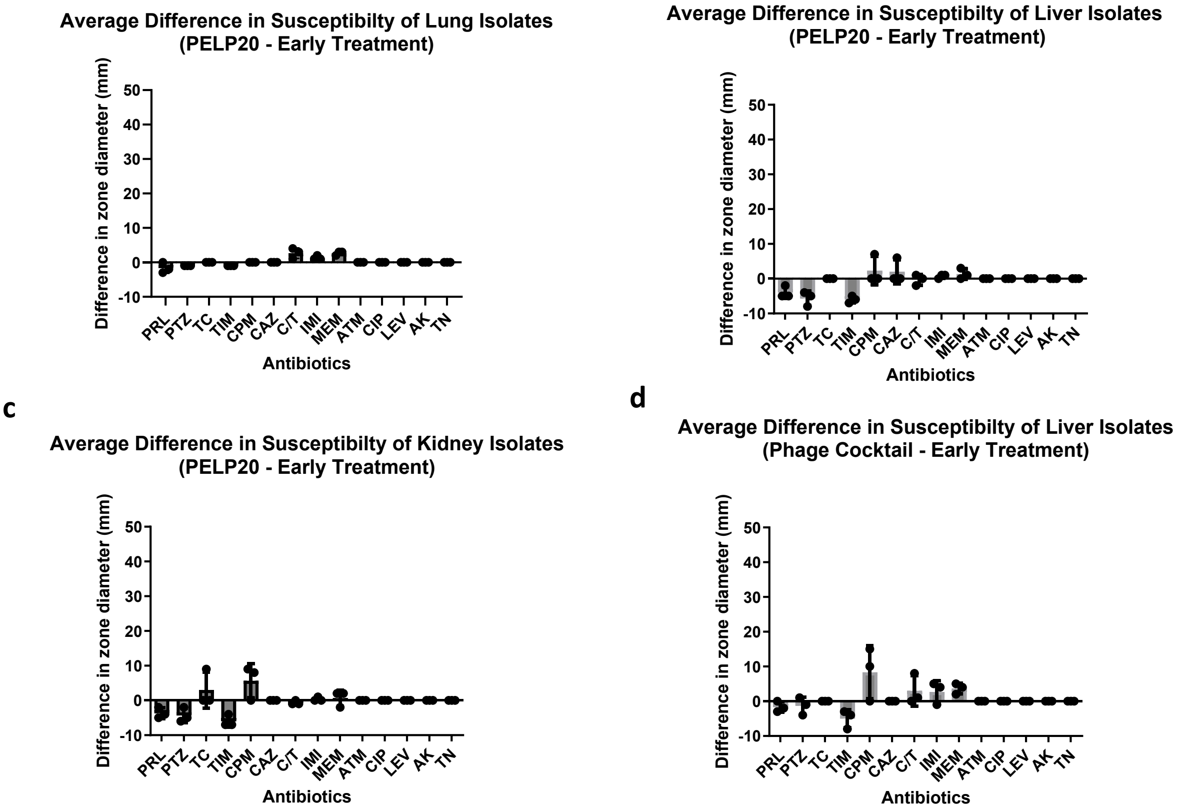

e

Isolates from Early Treated Phage *In Vivo* Experiment	Tissue	Resistance According to Zone Diameter (mm)		
		Sensitive	Intermediate	Resistant
Original B9		None	PTZ & TIM	PRL, TC, CPM, CAZ, C/T, IMI, MEM, ATZ, CIP, LEV, TN, AK
PELP20 Treated Mice	Lung 1	None	PTZ & TIM	PRL, TC, CPM, CAZ, C/T, IMI, MEM, ATZ, CIP, LEV, TN, AK
	Lung 2	None	PTZ & TIM	PRL, TC, CPM, CAZ, C/T, IMI, MEM, ATZ, CIP, LEV, TN, AK
	Lung 3	None	PTZ & TIM	PRL, TC, CPM, CAZ, C/T, IMI, MEM, ATZ, CIP, LEV, TN, AK
	Liver 1	None	PTZ	PRL, TC, TIM, CPM, CAZ, C/T, IMI, MEM, ATZ, CIP, LEV, TN, AK
	Liver 2	None	None	PRL, PTZ, TC, TIM, CPM, CAZ, C/T, IMI, MEM, ATZ, CIP, LEV, TN, AK
	Liver 3	None	PTZ	PRL, TC, TIM, CPM, CAZ, C/T, IMI, MEM, ATZ, CIP, LEV, TN, AK
	Kidney 1	None	PTZ	PRL, TC, TIM, CPM, CAZ, C/T, IMI, MEM, ATZ, CIP, LEV, TN, AK
	Kidney 2	None	None	PRL, PTZ, TC, TIM, CPM, CAZ, C/T, IMI, MEM, ATZ, CIP, LEV, TN, AK
	Kidney 3	None	PTZ	PRL, TC, TIM, CPM, CAZ, C/T, IMI, MEM, ATZ, CIP, LEV, TN, AK
Phage Cocktail Treated Mice	Liver 1	None	PTZ & MEM	PRL, TC, TIM, CPM, CAZ, C/T, IMI, ATZ, CIP, LEV, TN, AK
	Liver 2	None	PTZ	PRL, TC, TIM, CPM, CAZ, C/T, IMI, MEM, ATZ, CIP, LEV, TN, AK
	Liver 3	C/T	PTZ	PRL, TC, TIM, CPM, CAZ, IMI, MEM, ATZ, CIP, LEV, TN, AK

f

	Minimum Inhibitory Concentration (ug/ul)	
	Meropenem	Tobramycin
Input B9 isolate	>32	>256
Early PELP20 treated isolates		
Lung 1	32	256
Lung 2	>32	>256
Lung 3	32	256
Liver 1	12	>256
Liver 2	12	256
Liver 3	>32	>256
Kidney 1	16	256
Kidney 2	12	>256
Kidney 3	>32	256
Early Phage cocktail treated isolates		
Liver 1	>32	>256
Liver 2	16	>256
Liver 3	12	>256

Fold reduction in MIC
0
2
4
6
8
10

Supplementary Figure 5. Increased Antibiotic Susceptibility of Early Phage Treated Isolates Recovered From Systemic infection In Vivo Model.

Shows the difference in inhibition zone diameter in millimetres compared to the input *P. aeruginosa* isolate to a panel of antibiotics for isolates recovered from the a) lungs of early PELP20 treated mice b) liver of early PELP20 treated mice c) kidney of early PELP20 treated mice and d) liver of early phage cocktail treated mice. The mean with SD is indicated and n= 3 isolates tested per group. Black circles represent an individual isolate e) shows a summary of changes in resistance classification according to EUCAST breakpoints. Panel of antibiotics includes: Piperacillin (PRL), Piperacillin-tazobactam (PTZ), Ticarcillin (TC), Ticarcillin-clavulanic acid (TIM), Cefepime (CPM), Ceftazidime (CAZ), Ceftolozane-tazaobactam (C/T), Imipenem (IMI), Meropenem (MEM), Aztreonam (ATM), Ciprofloxacin (CIP), Levofloxacin (LEV), Amikacin (AK), Tobramycin (TN) f) shows the MIC as determine via E-test of tobramycin and meropenem. Dark blue squares show fold reduction in MIC, white show no change in MIC. Source data are provided as a Source Data file.

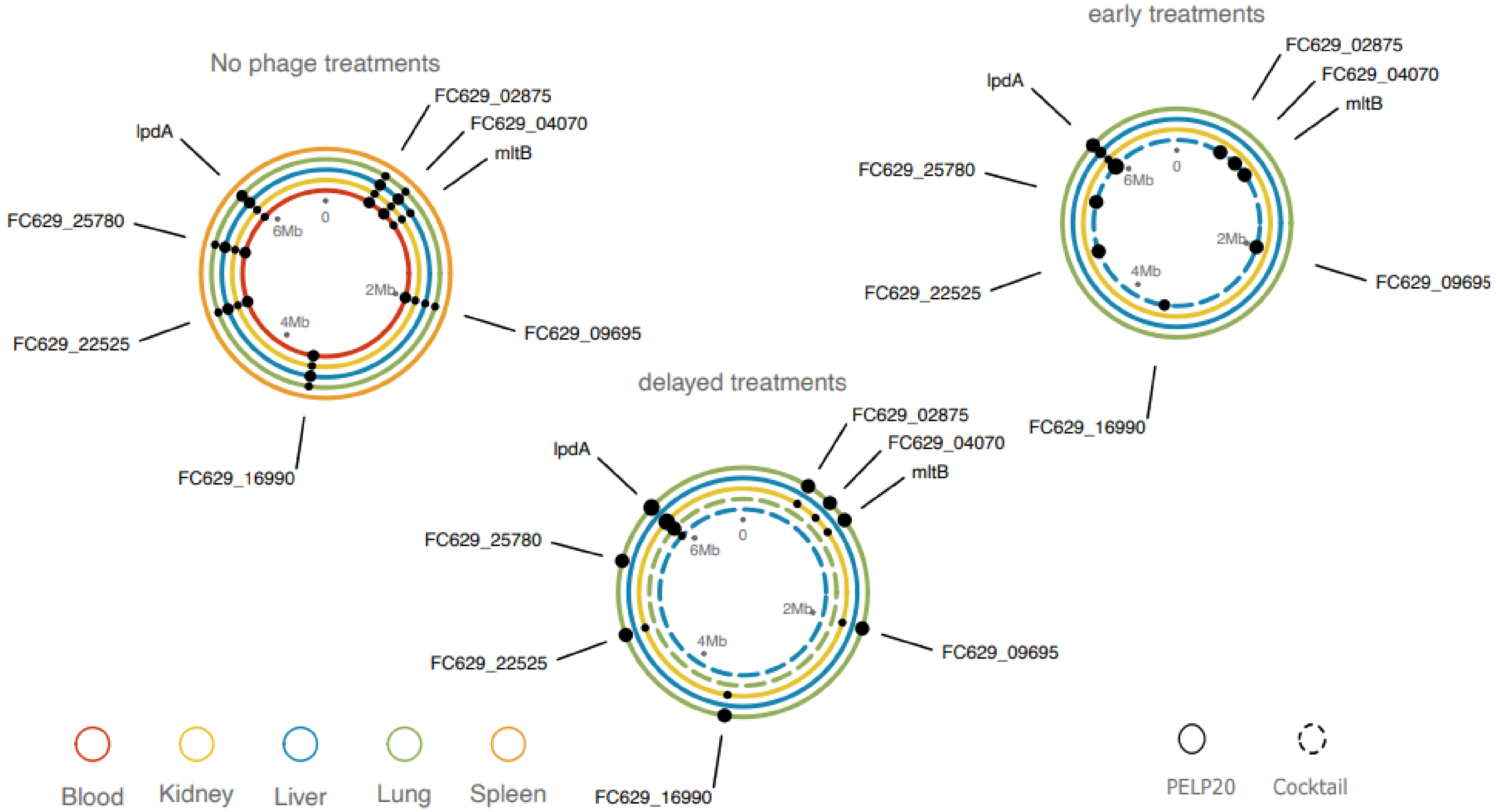

Supplementary Figure 6. Low frequency SNPs present in input B9 infection stock which have become fixed in the non-phage treated and phage treated *in vivo* adapted isolates. Shows the validated SNPS present in, non-phage treated isolates, early phage treated isolates and delayed phage treated isolates, that were present in the input B9 ancestor infection stock at low frequency. The lines represent isolates recovered from different tissues red=blood, yellow=kidney, blue=liver, green=lung, spleen=orange, and in the phage treated plots s solid line represents isolates recovered from PELP20 treated mice and dashed line represents isolates recovered from mice that received phage cocktail treatment. The dots represent a SNP, with the size of the dot corresponding with the number of replicates that contained each SNP. Source data are provided as a Source Data file and Supplementary data 2

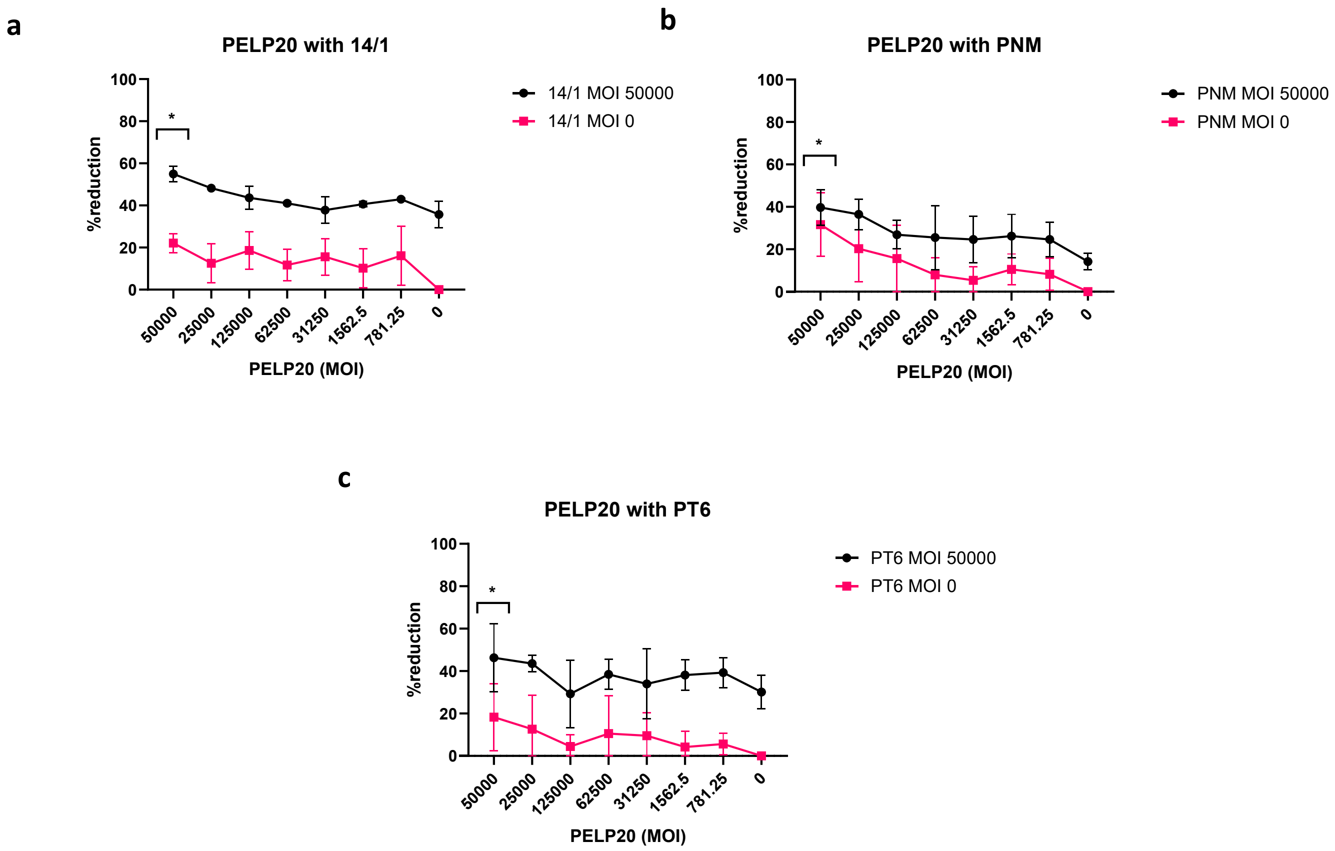

Supplementary Figure 7. Reduction of bacterial growth grown in the presence of different phage combinations. Shows the % reduction in bacterial growth (determined via OD 600) of *P. aeruginosa* strain B9 grown in the presence of different concentrations of PELP20 with different concentrations of a) 14/1 ($p= 0.0132$) b) PNM ($p= 0.0237$) and c) PT6 ($p= 0.0287$). * shows significance ($p<0.05$) of highest concentration of PELP20 grown in the presence or absence of another phage. The mean is indicated with the SD, black circles indicate either phage a) 14/1, b) PNM or c) PT6 at MOI 50000 and pink squares represent either phage a) 14/1, b) PNM or c) PT6 at 0 MOI. Statistics were performed via two-way ANOVA with Bonferroni correction post hoc test was performed when comparing more than 3 experimental groups. Source data are provided as a Source Data file.

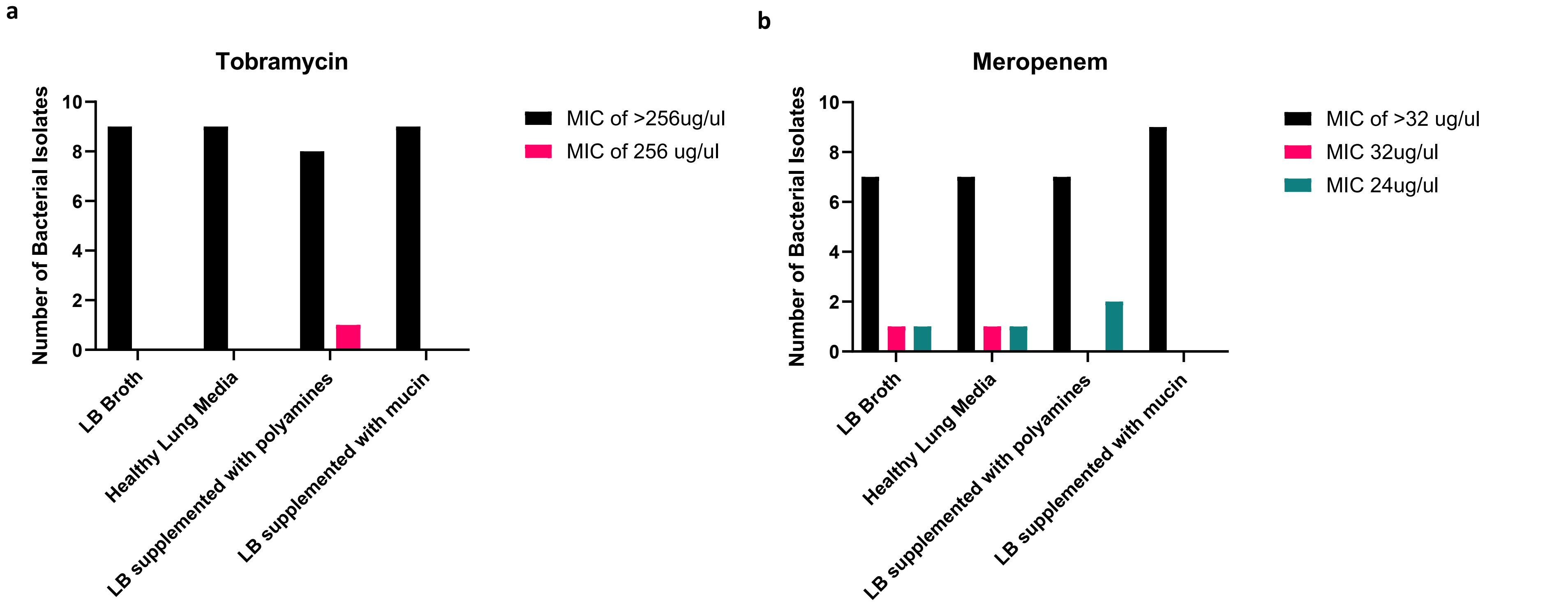

Supplementary Figure 8. Antibiotic sensitivity of isolates grown in lung environment conditions. a) Shows the number of isolates recovered from LB broth, healthy lung media, LB supplemented with polyamines and LB broth supplemented with mucin at 48hr with MIC of >256ug/ul and 256ug/ul of tobramycin. b) Shows the number of isolates recovered from LB broth, healthy lung media, LB supplemented with polyamines and LB broth supplemented with mucin at 48hr with MIC of >32ug/ul, 32ug/ul and 24ug/ul of meropenem. For each group 3 isolates in total were recovered from 3 different populations, therefore a total of 9 isolates per group were tested. Source data are provided as a Source Data file.
