# Supplementary material for: Exploiting lung adaptation and phage steering to clear pan-resistant Pseudomonas aeruginosa infections in vivo
Source: Nat Commun. 2024 Feb 20;15:1547. doi: 10.1038/s41467-024-45785-z (PMC10879199; doi:10.1038/s41467-024-45785-z)
Supplement: Supplementary file 3 — Description of Additional Supplementary Files [file 41467_2024_45785_MOESM3_ESM.pdf]

# <u>Description of Additional Supplementary Files</u>

**Supplementary Data 1 :** Full phage cocktail and single phage screening against 551 clinical isolates.

**Supplementary Data 2 :** Mutations identified in *P. aeruginosa* isolates using whole genome sequencing.
