# Supplementary material for: Exploiting lung adaptation and phage steering to clear pan-resistant Pseudomonas aeruginosa infections in vivo
Source: Nat Commun. 2024 Feb 20;15:1547. doi: 10.1038/s41467-024-45785-z (PMC10879199; doi:10.1038/s41467-024-45785-z)
Supplement: Supplementary file 6 — Reporting Summary [file 41467_2024_45785_MOESM6_ESM.pdf]

nature portfolio

Corresponding author(s): J L Fothergill

Last updated by author(s): 15/12/2023

Reporting Summary

Nature Portfolio wishes to improve the reproducibility of the work that we publish. This form provides structure for consistency and transparency in reporting. For further information on Nature Portfolio policies, see our Editorial Policies and the Editorial Policy Checklist.

Statistics

For all statistical analyses, confirm that the following items are present in the figure legend, table legend, main text, or Methods section.

n/a	Confirmed	
☐	☒	The exact sample size (*n*) for each experimental group/condition, given as a discrete number and unit of measurement
☐	☒	A statement on whether measurements were taken from distinct samples or whether the same sample was measured repeatedly
☐	☒	The statistical test(s) used AND whether they are one- or two-sided<br>*Only common tests should be described solely by name; describe more complex techniques in the Methods section.*
☒	☐	A description of all covariates tested
☐	☒	A description of any assumptions or corrections, such as tests of normality and adjustment for multiple comparisons
☐	☒	A full description of the statistical parameters including central tendency (e.g. means) or other basic estimates (e.g. regression coefficient) AND variation (e.g. standard deviation) or associated estimates of uncertainty (e.g. confidence intervals)
☐	☒	For null hypothesis testing, the test statistic (e.g. *F*, *t*, *r*) with confidence intervals, effect sizes, degrees of freedom and *P* value noted<br>*Give P values as exact values whenever suitable.*
☒	☐	For Bayesian analysis, information on the choice of priors and Markov chain Monte Carlo settings
☒	☐	For hierarchical and complex designs, identification of the appropriate level for tests and full reporting of outcomes
☒	☐	Estimates of effect sizes (e.g. Cohen's *d*, Pearson's *r*), indicating how they were calculated

Our web collection on statistics for biologists contains articles on many of the points above.

Software and code

Policy information about availability of computer code

Data collection	Illumina platform (MiSeq) to produce paired short reads (~250bp), Omega series V1.51
Data analysis	GraphPad Prism 7 software for Windows (GraphPad Inc, La Jolla, Calif) , R (version 4.0.4), igv

For manuscripts utilizing custom algorithms or software that are central to the research but not yet described in published literature, software must be made available to editors and reviewers. We strongly encourage code deposition in a community repository (e.g. GitHub). See the Nature Portfolio guidelines for submitting code & software for further information.

Data

Policy information about availability of data

All manuscripts must include a data availability statement. This statement should provide the following information, where applicable:

- Accession codes, unique identifiers, or web links for publicly available datasets
- A description of any restrictions on data availability
- For clinical datasets or third party data, please ensure that the statement adheres to our policy

The genetic sequences acquired during this study have been deposited into the National Center for Biotechnology Information database as a BioProject under accession number PRJEB67471. P. aeruginosa B9 reference genome can be accessed via GenBank accessions NZ_CP039988 and NZ_CP039989. Accession number for phage 14/1 is NC_011703 and sequences for PELP20 and PNM can be made available upon request. Source data are provided for this paper.

Research involving human participants, their data, or biological material

Policy information about studies with human participants or human data. See also policy information about sex, gender (identity/presentation), and sexual orientation and race, ethnicity and racism.

Reporting on sex and gender	Clinical isolates were sourced from previously published studies. These were Cazares et al., Nat Commun. 2020 Mar 13;11(1):1370. for the pan-resistant clinical isolate used in the in vivo studies. The isolates for phage screening came from the published studies, Hilliam et al., Eur Respir J. 2017 Apr 26;49(4):1602108, Shankar et al., FEMS Microbiol Lett. 2012 Sep;334(2):79-86. and Stewart et al., J Clin Microbiol. 2011 Mar;49(3):993-1003. The isolates from the Hilliam et al., publication originated from a clinical trial (Haworth et al., Am J Respir Crit Care Med. 2014 Apr 15;189(8):975-82).
Reporting on race, ethnicity, or other socially relevant groupings	The baseline characteristics from this trial were 43% male participants (57% female) and an average age of 59.3 years.
Population characteristics	Data is unavailable for all of these studies. The pan-resistant isolate was derived from an individual in Thailand suffering from acute respiratory infection. The screening clinical isolates derived from various studies but all from geographical locations witihin the United Kingdom.
Recruitment	Not applicable. No participants were recruited as part of this study
Ethics oversight	All isolates were from previously published papers therefore not applicable to this study

Note that full information on the approval of the study protocol must also be provided in the manuscript.

Field-specific reporting

Please select the one below that is the best fit for your research. If you are not sure, read the appropriate sections before making your selection.

☒ Life sciences ☐ Behavioural & social sciences ☐ Ecological, evolutionary & environmental sciences

For a reference copy of the document with all sections, see nature.com/documents/nr-reporting-summary-flat.pdf

Life sciences study design

All studies must disclose on these points even when the disclosure is negative.

Sample size	Sample size determination was performed as previously described, informed from previous work comparing P. aeruginosa infection density in mice Fothergill, J. L. et al, Nat. Commun. 5, 4780 (2014) and sample sizes of 10 were chosen to obtain significance. Our pilot experiments (Supplementary figure 1 and 2) showed 100% survival for isolate B9 in 6-8 week old BALB/c mice, with a sample size of n=5 due to ethics considerations.
Data exclusions	No data was excluded
Replication	Data in the following figures are from two independent experiments: Figure 2a-e, Figure 3a-e, Figure 9a-e, Figure 10a-e<br>Data in the following figures are from single experiments: Figure 2f, Figure 3f, Supplementary figure 1, 2, 3<br>Data from the following figures are from a single experiment with three biological replicates. Fig 4, 5, 6, 7, 8b-d<br>Figure 8a (sequencing data) three biological replicates were sequenced for each treatment group.
Randomization	Mice were randomized to cages on arrival at the University of Liverpool animal facility, by technical staff with no role in study design. At study onset, random allocation of unique cage I.D. numbers to experimental groups was performed by the research team. All mice used were female BALB/c of 6-8 weeks of age. Within each experiment, all mice used were within 2 weeks of age of each other.
Blinding	No blinding was performed due to experimental procedures on animals carried out by one researcher.

Reporting for specific materials, systems and methods

We require information from authors about some types of materials, experimental systems and methods used in many studies. Here, indicate whether each material, system or method listed is relevant to your study. If you are not sure if a list item applies to your research, read the appropriate section before selecting a response.

Materials & experimental systems

n/a	Involved in the study
☒	☐ Antibodies
☒	☐ Eukaryotic cell lines
☒	☐ Palaeontology and archaeology
☐	☒ Animals and other organisms
☒	☐ Clinical data
☒	☐ Dual use research of concern
☒	☐ Plants

Methods

n/a	Involved in the study
☒	☐ ChIP-seq
☒	☐ Flow cytometry
☒	☐ MRI-based neuroimaging

Animals and other research organisms

Policy information about studies involving animals; ARRIVE guidelines recommended for reporting animal research, and Sex and Gender in Research

Laboratory animals	6-8week old, female BALB/c were purchased from Charles River Laboratories (Charles River, UK). The animals were housed in the animal facilities under the following conditions: Temperature: 21-23 degrees Celsius Humidity: 55-65% Cages: Individually ventilated cages (IVC) from Technoplast (GMS00), 12hr light-dark cycle. Water supply: Automating watering providing reverse osmosis water sterilised by UV radiation. Enrichment: Nesting material, balcony, dome home, handling tunnel.
Wild animals	Study did not involve wild animals
Reporting on sex	Experiments were all performed on female BALB/c mice to generate reliable data and maintain statistical power.
Field-collected samples	Study did not involve samples collected from the field
Ethics oversight	This study was performed in strict accordance with UK Home Office guidelines. Animal experiments were performed at the University of Liverpool and were approved by the University of Liverpool Animal Welfare Research Body (AWERB).

Note that full information on the approval of the study protocol must also be provided in the manuscript.

Plants

Seed stocks	*Report on the source of all seed stocks or other plant material used. If applicable, state the seed stock centre and catalogue number. If plant specimens were collected from the field, describe the collection location, date and sampling procedures.*
Novel plant genotypes	*Describe the methods by which all novel plant genotypes were produced. This includes those generated by transgenic approaches, gene editing, chemical/radiation-based mutagenesis and hybridization. For transgenic lines, describe the transformation method, the number of independent lines analyzed and the generation upon which experiments were performed. For gene-edited lines, describe the editor used, the endogenous sequence targeted for editing, the targeting guide RNA sequence (if applicable) and how the editor was applied.*
Authentication	*Describe any authentication procedures for each seed stock used or novel genotype generated. Describe any experiments used to assess the effect of a mutation and, where applicable, how potential secondary effects (e.g. second site T-DNA insertions, mosiacism, off-target gene editing) were examined.*
